# Trends in Factors Affecting Pregnancy Outcomes Among Women With Type 1 or Type 2 Diabetes of Childbearing Age (2004–2017)

**DOI:** 10.3389/fendo.2020.596633

**Published:** 2021-02-22

**Authors:** Mariangela Gaudio, Nicoletta Dozio, Michael Feher, Marina Scavini, Amelia Caretto, Mark Joy, Jeremy Van Vlymer, William Hinton, Simon de Lusignan

**Affiliations:** ^1^ Department of Clinical and Experimental Medicine, University of Surrey, Guildford, United Kingdom; ^2^ International Medical Doctor Program, Vita-Salute San Raffaele University, Milan, Italy; ^3^ Diabetes Research Institute, San Raffaele Scientific Institute, Milan, Italy; ^4^ Nuffield Department of Primary Care Health Medical Sciences Division, University of Oxford, Oxford, United Kingdom; ^5^ Royal College of General Practitioners (RCGP) Research and Surveillance Centre (RSC), London, United Kingdom

**Keywords:** medical records system, computerized, diabetes mellitus, diabetes mellitus, type 1, diabetes mellitus, type 2, pregnancy, high risk, general practice

## Abstract

**Aim:**

To describe trends in modifiable and non-modifiable unfavorable factors affecting pregnancy outcomes, over time (years 2004–2017), in women with diabetes of childbearing age from an English primary care perspective.

**Methods:**

We identified women with diabetes aged 16–45 years from the Royal College of General Practitioners (RCGP) Research and Surveillance Centre (RSC) network, an English primary care sentinel database. Repeated annual cross-sectional analyses (2004–2017) assessed the prevalence of unfavorable factors for pregnancy, such as obesity, poor glycaemic control, microalbuminuria, hypertension, use of medications for treating diabetes, and associated comorbidities not recommended for pregnancy.

**Results:**

We identified 3,218 women (61.5% with Type 2 diabetes) in 2004 and 6,657 (65.0% with Type 2 diabetes) in 2017. The proportion of women with ideal glycaemic control for conception (HbA1c<6.5%) increased over time, in patients with Type 1 diabetes from 9.0% (7.1%–11.0%) to 19.1% (17.2%–21.1%), and in those with Type 2 diabetes from 27.2% (24.6%–29.9%) to 35.4% (33.6%–37.1%). The proportion of women with Type 2 diabetes prescribed medications different from insulin and metformin rose from 22.3% (20.5%–24.2%) to 27.3% (26.0%–28.6%).

In 2017, 14.0% (12.6%–15.4%) of women with Type 1 and 30.7% (29.3%–32.0%) with Type 2 diabetes were prescribed angiotensin-modulating antihypertensives or statins. We captured at least one unfavorable factor for pregnancy in 50.9% (48.8%–52.9%) of women with Type 1 diabetes and 70.7% (69.3%–72.0%) of women with Type 2 diabetes. Only one third of women with Type 1 diabetes (32.2%, 30.3%–34.0%) and a quarter of those with Type 2 diabetes (23.1%, 21.9%–24.4%) were prescribed hormonal contraception. Contraception was prescribed more frequently to women with unfavorable factors for pregnancy compared to those without, however, the difference was significant only for women with Type 1 diabetes.

**Conclusions:**

Despite significant improvements in general diabetes care, the majority of women with Type 1 or Type 2 diabetes have unfavorable, although mostly modifiable, factors for the start of pregnancy. Good diabetes care for women of childbearing age should include taking into consideration a possible pregnancy.

## Introduction

Effective pre-pregnancy care for women with Type 1 or Type 2 diabetes reduces adverse pregnancy outcomes (1, 2). Several studies, including the National Pregnancy in Diabetes (NPID) Audit (3), have unequivocally demonstrated that the risks of both obstetric [pre-eclampsia, polyhydramnios, preterm delivery, shoulder dystocia, and Caesarean section (4)] and foetal [congenital malformation, macrosomia, and stillbirth (5)] complications can be reduced by the optimization of several aspects of diabetes care, actively considering the possibility of pregnancy.

In the United Kingdom, the 2015 National Institute for Health and Care Excellence (NICE) guidelines on diabetes and pregnancy set out how pre-pregnancy care should be integrated into routine diabetes care from puberty onward. Conception should ideally take place when HbA1c is below 48 mmol/mol (6.5%) and be discouraged if above 86 mmol/mol (10%). Folic acid at the dose of 5 mg per day should be prescribed before conception. Several medications commonly prescribed for the treatment of diabetes [(e.g., sodium-glucose co-transporter-2 (SGLT2) inhibitors, glucagon-like peptide-1 (GLP1) analogs, dipeptidyl peptidase 4 inhibitors (DPP4i)] and of comorbidities (e.g., statins and renin-angiotensin modulating antihypertensives) should be discontinued before conception or replaced as soon as pregnancy is confirmed (6) due to potential harm or unknown effects on the foetus. Women with diabetes should primarily be counselled about contraception, since in this population of women still the majority of pregnancies are unplanned (7). The American Diabetes Association’s (ADA) recommendations are consistent with NICE: “The risk of an unplanned pregnancy outweighs the risk of any given contraception” (8). The care of young women with diabetes, especially those with Type 2 diabetes, is likely to take place more and more in a primary care setting, rather than in specialist centres. Therefore, it is imperative that the importance of a specific approach to the management of women of childbearing age is transferred also within these care teams.

We carried out this study to estimate the prevalence over time of factors that may affect pregnancy outcomes in women with Type 1 or Type 2 diabetes.

## Subjects, Material, and Methods

### Background About UK General Practice as a Comprehensive Data Source

The UK general practice system is a registration-based system, for every citizen who wishes to access the service is registered with a single practice. Medical records have been computerized since the 1990s with electronic transfer of records between practices (9). Prescribing is almost universally done from computerized medical record (CMR) systems, which also have direct electronic links to clinical pathology to ensure capture of laboratory results. Since 2004, a pay-for-performance (P4P) scheme, [Quality and Outcome Framework (QOF)], has been introduced for chronic diseases, including diabetes, ensuring at least yearly monitoring of patients (10) and offering the opportunity to analyse changes of care patterns overtime.

The Royal College of General Practitioners (RCGP) Research and Surveillance Centre (RSC) is a long-established general practice sentinel network (11, 12), which comprises CMR data from general practices distributed across England. The network is continuously recruiting new practices and its national representativeness and suitability for research compared with official national databases was demonstrated by Hinton W, McGovern A, Coyle R, et al. (13).

### Study Population

We included in our analysis women aged between 16 and 45 years with a diagnosis of either Type 1 or Type 2 diabetes (**Supplementary Table 1**), registered within RCGP RSC network. The RCGP RSC database contained data on 2,026,150 patients registered at the end of December 2017 of whom 316,461 where females aged between 16 and 45 years. The dataset used for this study comprised yearly collected data from 2004 to end of 2017, including demographics, coded diagnostic, laboratory test, and prescription data for women in our study age-band with diabetes (14). We excluded women who were pregnant during the observation period for the duration of pregnancy. Likewise, we excluded women with menopause, either premature ovarian failure or surgical, from the date of the documented menopause.

### Variables of Interest

Diagnosis and type of diabetes were identified using coded data from the CMR system, using a validated algorithm. Key data were recorded using the Read terminology in UK primary care and identified using an established ontological process (15, 16).

Demographic variables included in the study were age, ethnicity (self-reported), and socio-economic status. Socioeconomic status was derived from the English index of multiple deprivation (IMD) and divided into quintiles, where 1 is the most and 5 the least deprived quintile. We extracted the latest recorded glycated haemoglobin value (HbA1c) in each year, reported as percent of total haemoglobin according to Diabetes Control Complications Trial (DCCT), but also stating the equivalent International Federation of Clinical Chemists (IFCC) units. Glycaemic control was categorized as follows: ideal HbA1c < 6.5% (48 mmol/mol); good [6.5%–7.5% (48–58 mmol/mol)]; moderate [7.5%–8.5% (58–69 mmol/mol); poor [≥8.5% (69 mmol/mol)]. We reported the presence of microalbuminuria as a single measurement of elevated Urine albumin to creatinine ratio (UACR). Evidence of hypertension was defined as code for hypertension or SBP ≥ 140 mmHg or DBP ≥ 90 mmHg, regardless of whether prescribed antihypertensive medication. Smoking status was categorized as current smoker, ex-smoker and never smoked. BMI was reported in kg/m^2^ and obesity was defined as unfavorable factor when BMI ≥ 30.

We analyzed the use of medications for the treatment of diabetes and associated comorbidities, which are not recommended during pregnancy. For diabetes these included: sulfonylureas, DPP4 inhibitors, meglitinide, SGLT-2 inhibitors, GLP-1 receptor agonists, alpha-glucosidase inhibitors (acarbose), and pioglitazone. We looked at the following medications for comorbidities: ACE inhibitors, angiotensin receptor blockers (ARBs) and statins. We analyzed the prescription of contraception including oral combined and progesterone only preparations, injectable, implantable, and intrauterine device, i.e., all contraceptive methods requiring medical prescription.

### Data Analysis

We carried out yearly repeated cross-sectional analyses from 2004 to 2017. Descriptive statistics were used to summarize the clinical features of the study population (HbA1c, BMI, medication use, etc.). We used Pearson’s Chi-square test to compare proportions, comparisons with p values < 0.001 were considered significant.

We computed a cumulative risk score of unfavorable factors should a pregnancy occur. We included in this score each of the followings: BMI ≥ 30 kg/m^2^; HbA1c ≥ 8.5% (69 mmol/mol); presence of microalbuminuria; diagnosis of hypertension; use of statins, ACE inhibitors or ARBs; use of at least one diabetes medication other than metformin and insulin (for Type 2 diabetes patient only) (17).

All statistical analyses were performed using R 3.5.1 statistical software.

### Ethical Approval

Data were pseudonymized at the point of extraction and no clinically identifiable information was available to researchers. The study was classified as clinical audit by the Heath Research Authority (HRA)/Medical Research Council (MRC) “Is my study research?” tool (http://www.hra-decisiontools.org.uk/research/) (18). The study protocol was approved by the RCGP RSC study approval committee on March 27^th^, 2018.

## Results

### Demographic and Social Factors

There were 316,461 women of childbearing age registered with RCGP RSC practices in 2004 and 465,898 in 2017 (**Supplementary Table 1**). Of these women, 3,218 (1.0%) had diabetes in 2004 and 6,657 (1.4%) in 2017. Most of the women had Type 2 diabetes (61.5% in 2004 and 65.0% in 2017).

Women with Type 1 diabetes were younger than those with Type 2 diabetes across the years under investigation [mean age: 2004: 30.8 years (SD 7.9) vs. 37.5 years (SD 6.1); 2017: 31.0 years (SD 7.9) vs. 37.7 years (SD 6.3)]. Furthermore, women living in the most deprived areas (IMD quintile 1) were more likely to have Type 2 than Type 1 diabetes. The majority of women had their smoking status recorded (in 2017 95.5% in Type 1 diabetes and 97.1% Type 2 diabetes), with the prevalence of current smokers reducing over time [Type 1 diabetes: 2004: 29.0% (26.5%–31.6%), 2017: 17.6% (16.1%–19.2%); Type 2 diabetes: 2004: 27.0% (21.1%–29.0%), 2017: 19.5% (18.3%–20.7%), *p* < 0.001] (**Table 1**).

**Table 1:** Characteristics of women with diabetes either Type 1 or Type 2 diabetes in 2004, 2011, and 2017.

Demographics	Type 1 diabetes				Type 2 diabetes			
	2004 N = 1,240	2011 N = 1,740	2017 N = 2,332	χ^2^ *test p-value*	2004 N = 1,978	2011 N = 3,261	2017 N = 4,325	χ^2^ *test p-value*
Age				*p* = 0.106				*p* = 0.1
<20	126(10.2)	159(9.1)	218(9.3)		20(1.0)	39(1.2)	47(1.1)	
20–24	179(14.4)	281(16.1)	334(14.3)		71(3.6)	89(2.7)	159(3.7)	
25–29	221(17.8)	293(16.8)	441(18.9)		148(7.5)	250(7.7)	331(7.7)	
30–34	270(21.8)	352(20.2)	485(20.8)		264(13.3)	448(13.7)	599(13.8)	
35–39	244(19.7)	316(18.2)	472(20.2)		569(28.8)	827(25.4)	1104(25.5)	
40–45	200(16.1)	339(19.5)	382(16.4)		906(45.8)	1608(49.3)	2085(48.2)	
IMD Quintile				*p =* 0.025				*p* = 0.041
1 (most deprived)	206(17.4)	291(17.7)	468(20.9)		487(25.5)	818(26.4)	1240(29.6)	
2	204(17.2)	294(17.9)	436(19.4)		388(20.6)	647(20.9)	849(20.2)	
3	234(19.3)	313(19.0)	385(17.2)		336(17.8)	567(18.3)	737(17.6)	
4	266(22.5)	368(22.4)	441(19.7)		354(18.8)	571(18.4)	729(17.4)	
5 (least deprived)	273(23.1)	379(23.0)	514(22.9)		328(17.4)	492(15.9)	638(15.2)	
Ethnicity				*p* < 0.001				*p* < 0.001
White	709(57.2)	1258(72.3)	1577(67.6)		1050(53.1)	1815(55.7)	2316(53.5)	
Asian	20(1.6)	95(5.5)	181(7.8)		247(12.5)	583(17.9)	914(21.1)	
Black	27(2.2)	37(2.1)	88(3.8)		97(4.9)	264(8.1)	356(8.2)	
Mixed	6(0.5)	14(0.8)	34(1.5)		23(1.2)	45(1.4)	85(2.0)	
Other	6(0.5)	13(0.7)	27(1.2)		13(0.7)	42(1.3)	70(1.6)	
Smoking habit				*p* < 0.001				*p* < 0.001
Never smoked	498(40.2)	761(43.7)	1123(48.2)		813(41.1)	1250(38.3)	1888(43.7)	
Active smoker	360(29.0)	434(24.9)	411(17.6)		535(27.0)	906(27.8)	843(19.5)	
Ex-smoker	275(22.2)	475(27.3)	692(29.7)		500(25.3)	1004(30.8)	1470(34.0)	
Unknown smoking status	107(8.6)	70(4.0)	106(4.5)		130(6.6)	101(3.1)	124(2.9)	

Data are presented as n (%). IMD, Index of Multiple Deprivation.

### Diabetes Control

**Figure 1:**
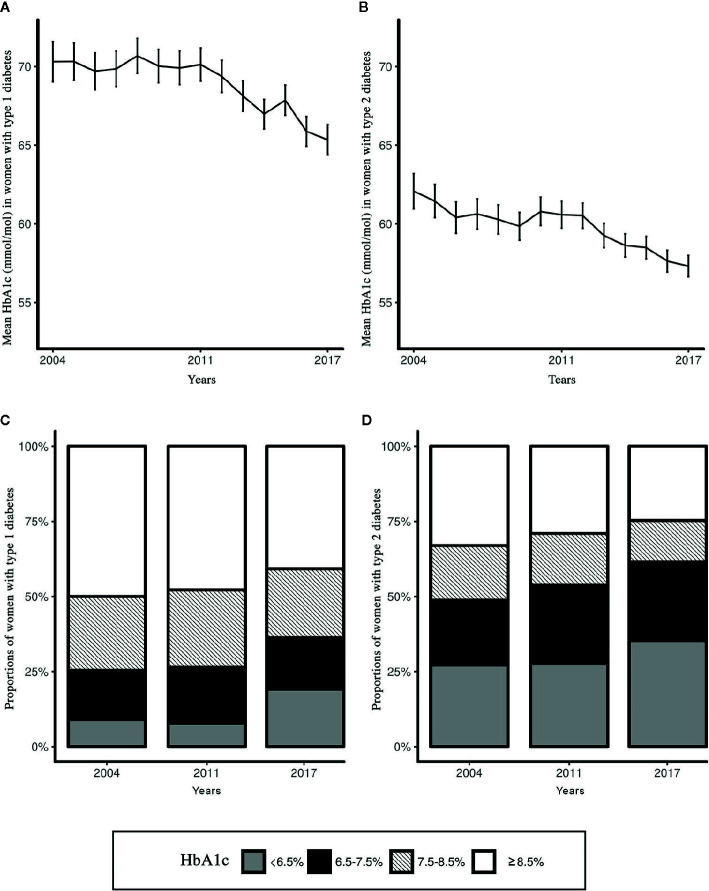
Trends over time (years 2004–2017) of glucose control of women of childbearing age with diabetes. **(A)** Mean HbA1c (mmol/mol) women with Type 1 diabetes; **(B)** Mean HbA1c (mmol/mol) in women with Type 2 diabetes; **(C)** Proportions of women with Type 1 diabetes by HbA1c category (years 2004, 2011, and 2017); **(D)** Proportions of women with Type 2 diabetes by HbA1c category (years 2004, 2011, and 2017).

Between 2004 and 2017 in women with Type 1 diabetes, mean HbA1c improved from 8.6% to 8.1% [70 mmol/mol (SD 18) to 65 mmol/mol (SD 19)], and for those with Type 2 diabetes from 7.8% to 7.4% [62 mmol/mol (SD 19) to 57 mmol/mol (SD 19)] (**Figures 1A, B**). During the observation period, the proportion of women with Type 1 diabetes with ideal glucose control in terms of HbA1c increased from 9.0% (7.0%–11.0%) to 19.1% (17.2%–21.1%), p < 0.001). By comparison, among women with Type 2 diabetes the proportion with ideal glucose control increased from 27.2% (24.6%–29.9%) to 35.4% (33.6%–37.1%) (p < 0.001). The proportion of women with poor glucose control decreased over time both in patients with Type 1 diabetes [from 50.0% (46.4%–53.6%) to 40.8% (38.4%–43.2%), p < 0.001)], and patients with Type 2 diabetes [from 33.1% (30.3%–35.9%) to 24.7% (23.2%–26.2%), p < 0.001) (**Figures 1C, D** and **Table 2**). Women with Type 1 diabetes having a record of microalbuminuria decreased from 25.1% (22.7%–27.5%) to 23.7% (22.0%–25.4%) (*p* < 0.001), while among women with Type 2 diabetes the proportion having a record of microalbuminuria increased from 22.8% (20.9%–24.6%) to 30.1% (28.7%–3.5%) (*p* < 0.001).

**Table 2:** Women with either Type 1 or Type 2 diabetes by HbA1c category.

	Type 1 diabetes				Type 2 diabetes			
	2004 N = 788	2011 N = 1,177	2017 N = 1,579	χ^2^ *test p-value*	2004 N = 1,098	2011 N = 1,949	2017 N = 3,041	χ^2^ *test p-value*
**HbA1c category**				P < 0.001				P < 0.001
<6.5% (ideal)	71 (9.0)	93 (7.9)	302 (19.1)		299 (27.2)	539 (27.7)	1075 (35.4)	
6.5%–7.5%	129 (6.4)	218 (18.5)	271 (17.2)		236 (21.5)	509 (26.1)	797 (26.2)	
7.5%–8.5%	194 (24.6)	303 (25.7)	362 (22.9)		200 (18.2)	335 (17.2)	418 (13.7)	
≥8.5% (poor)	394 (50.0)	563 (47.8)	644 (40.8)		363 (33.1)	566 (29.0)	751 (24.7)	

Data are presented as n (%).

### Comorbidities and Medications

Compared to Type 1 diabetes, women with Type 2 diabetes were more likely to be obese. The prevalence of obesity increased over time for all women with diabetes [Type 1 diabetes: 2004: 19.8% (17.0%–22.6%), 2017: 26.0% (23.7%–28.3%); Type 2 diabetes: 2004: 66.2% (63.6%–68.8%), 2017: 68.1% (66.3%–69.8%), *p* < 0.001]. The prevalence of women with evidence of hypertension decreased significantly both in women with Type 1 or Type 2 diabetes, being higher in the latter group [Type 1 diabetes: 2004: 16.3% (14.3%–18.4%), 2017: 12.3% (11.0%–13.7%); Type 2 diabetes: 2004: 28.6% (26.6%–30.6%), 2017: 22.3% (21.1%–23.6%), *p* < 0.001].

The use of medications for the treatment of Type 2 diabetes other than metformin and insulin increased from 22.3% (20.5%–24.2%) to 27.3% (26.0%–28.6%) (*p* < 0.001). Women using at least one medication acting on the renin angiotensin system or statins decreased from 21.3% (19.0%–23.6%) to 14.0% (12.6%–15.4%) (*p* < 0.001) and from 34.6% (32.5%–36.7%) to 30.7% (29.3%–32.0%) (*p* < 0.001) in women with Type 1 and Type 2 diabetes, respectively (**Table 3**).

**Table 3:** Clinical characteristics of women with diabetes either Type 1 or Type 2 diabetes in 2004, 2011, and 2017.

Clinical Characteristics	Type 1 diabetes				Type 2 diabetes			
	2004 N = 1,240	2011 N = 1,740	2017 N = 2,332	χ^2^ *test p-value*	2004 N = 1,978	2011 N = 3,261	2017 N = 4,325	χ^2^ *test p-value*
BMI*				*p* < 0.001				*p* = 0.011
Underweight	19(2.4)	24(2.0)	40(2.9)		12(1.0)	11(0.5)	18(0.7)	
Normal weight	366(46.2)	500(42.6)	578(41.3)		143(11.6)	222(10.2)	301(11.0)	
Overweight	251(31.7)	359(30.6)	417(29.8)		261(21.2)	448(20.6)	552(20.2)	
Obese	157(19.8)	292(24.9)	364(26.0)		816(66.2)	1495(68.7)	1858(68.1)	
Hypertension	202(16.3)	248(14.3)	288(12.3)	*p* < 0.001	566(28.6)	853(26.2)	966(22.3)	*p* < 0.001
Microalbuminuria	311(25.1)	590(33.9)	552(23.7)	*p* < 0.001	450(22.8)	1171(35.9)	1302(30.1)	*p* < 0.001
Medications not recommended for the use in pregnancy	264(21.3)	346(19.9)	327(14.0)	*p* < 0.001	684(34.6)	1227(37.6)	1326(30.7)	*p* < 0.001
ACE inhibitors/ARBs	163(13.1)	185(10.6)	188(8.1)	*p* < 0.001	428(21.6)	692(21.2)	723(16.7)	*p* < 0.001
Statins	163(13.1)	263(15.1)	231(9.9)	*p* < 0.001	494(25.0)	958(29.4)	969(22.4)	*p* < 0.001
Diabetes medication other than metformin and insulin	0(0.0)	8(0.5)	8(0.3)	*p* = 0.069	442(22.3)	730(22.4)	1180(27.3)	*p* < 0.001
Women with at least one unfavorable factor for pregnancy	671(54.1)	1080(62.1)	1186(50.9)	*p* < 0.001	1352(68.4)	2341(71.8)	3057(70.7)	*p = 0.29*

Data are presented as n (%).

ARBs, Angiotensin Receptor Blockers.

*Missing data.

### Unfavorable Factors for Pregnancy

**Figure 2:**
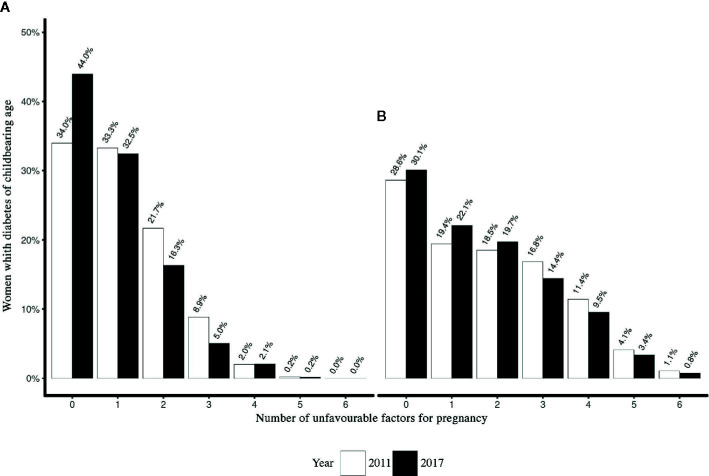
Cumulative score for factors considered to be unsafe in the case of pregnancy for women of childbearing age with **(A)** Type 1 diabetes and **(B)** Type 2 diabetes in 2011 and 2017.

We compared proportions of women with unfavorable factors for pregnancy in 2011 and 2017, according to diabetes type (**Figure 2**). Overall, women with Type 2 diabetes had more unfavorable factors when compared to those with Type 1 diabetes. The proportion of women with Type 1 diabetes who had at least one unfavorable factor decreased by 18% [2011: 62.1% (59.8%–64.4%) vs. 2017: 50.9% (48.8%–52.9%) (*p* < 0.001)], while that of women with Type 2 diabetes remained unchanged and accounted for approximately two thirds of the cases [2011: 71.8% (70.2%–73.3%) vs. 2017: 70.7% (69.3%–72.0%), (p = 0.29)].

### Contraception

Prescribing of contraception was higher among women with Type 1 diabetes than with Type 2 diabetes, with no changes across all years under observation [Type 1 diabetes: 2004: 33.8% (31.2%–36.5%), 2017: 32.2% (30.3%–34.0%), p = 0.323; Type 2 diabetes: 2004: 22.5% (20.7%–24.4%), 2017: 23.1% (21.9%–24.4%), *p* < 0.571].

Women with Type 1 diabetes with at least one unfavorable factor were prescribed contraception more frequently than those without (2011: 37.2% vs. 29.1%, *p* = 0.001; 2017: 36.4% vs. 28.6%, *p* < 0.001). Women with Type 2 with at least one unfavorable factor were prescribed contraception more frequently than those without in 2017 (24.5% vs. 20.0%, *p* = 0.002) and in 2011 (26.7% vs. 23.3%, *p* = 0.057), although not significantly (**Table 4**).

**Table 4:** Difference in contraception patterns of women with either Type 1 or Type 2 diabetes with at least one unfavorable factor in 2011 and 2017*.

Year		Type 1 diabetes			Type 2 diabetes		
		None unfavorable factor	At least one	χ^2^ *test p-value*	None unfavorable factor	At least one	χ^2^ *test p-value*
2011	N	536	1083		842	2219	
	Prescribed contraception (%)	156 (29.1)	403 (37.2)	0.001	196 (23.3)	593 (26.7)	0.057
2017	N	953	1216		1199	2898	
	Prescribed contraception (%)	273 (28.6)	443 (36.4)	<0.001	240 (20.0)	709 (24.5)	0.002

Data are presented as n (%).

*Missing data.

## Discussion

### Main Findings

Over the 13-year study period, there were significant improvements in several aspects of diabetes care in women of childbearing age, which might result from implementation of general guidelines, availability of newer medications for treating Type 2 diabetes, possibly driven by the pay-per performance scheme.

However, there is no evidence, from our data, that the reproductive potential of women is taken into consideration when making therapeutic choices for the management of their diabetes or associated comorbidities. In facts, more than half of the women with Type 1 diabetes and 70% of those with Type 2 diabetes have at least one unfavorable, although potentially modifiable, factor in the case of an unplanned pregnancy.

The reduction of an unhealthy lifestyle factor, such as smoking, reflects a proactive management by the health care system and/or a change in the social attitude about smoking. Nevertheless, one in five of the women with diabetes of childbearing age were still smoking. We documented an improvement in glucose control, hypertension management, and microalbuminuria; however, the majority of women with Type 1 diabetes were still not achieving an “ideal” HbA1c. The beneficial effects on glucose control brought to women with Type2 diabetes by the newer medications should be weighed against their undetermined risk for the foetus in case of a pregnancy.

Women with Type 2 diabetes were more likely to be obese and have hypertension or an abnormal lipid profile. Accordingly, they were prescribed more often drugs for the treatment of comorbidities that are not recommended for use in pregnancy (ACE inhibitors, ARBs, or statins) compared to women with Type 1; though we documented a reduction in the prescription of these medications between 2004 and 2017 in both types of diabetes. The high prevalence of obesity, especially among women with type 2 diabetes, is of concern, since obesity itself, independently of diabetes, is associated with negative pregnancy outcomes. The relatively high frequency of microalbuminuria in both patients with either Type 1 or Type 2 diabetes may be, at least in part, accounted for by the fact that we counted as having microalbuminuria patients with a single measurement of elevated UACR, rather than sustained elevated UACR, as recommended by guidelines (i.e., repeated UACR measurements). As for the high rate of microalbuminuria observed in women with Type 2 diabetes, we may hypothesize that is associated to the higher rate of hypertension and/or to a longer duration of disease than documented because of a delay in diabetes diagnosis.

Only one third of the women with Type 1 diabetes and one quarter of those with Type 2 diabetes were prescribed contraception by their GPs. Women with Type 1 diabetes with unfavorable factors were significantly more likely to be prescribed contraception than those without. Although, the same was also observed in 2017 among women with Type 2 diabetes, this was not significantly.

### Comparison With the Literature

The majority of studies looking at the effects of pre-pregnancy care for women with diabetes collected data at their presentation to the antenatal clinic, i.e., when already pregnant. These studies have highlighted a high proportion of unplanned pregnancies for both women with Type 1 diabetes who usually attend secondary care/specialist clinic, and women with Type 2 diabetes, usually seen only in general practice (19). Compared to an Italian study analyzing data from specialist clinics, attended by women with both Type 1 and Type 2 diabetes, we reported a more frequent use of medications not recommended for conception and pregnancy, such as statins and ACE inhibitors/ARBs, as well as a more aggressive management of their diabetes in terms of prescribed medications (17). The 2015 NPID audit compared with the 2002/2003 Confidential Enquiry into Maternal and Child Health (CEMACH), performed in antenatal clinic, reported an improvement in glucose control in women with Type 2 diabetes, but not in those with Type 1 diabetes (20). Other authors reported the use of medications not recommended for use in pregnancy in women with diabetes, finding no association with the concomitant prescription of contraception (21).

Educational interventions aimed at healthcare professionals and patients have shown improvements in pregnancy preparation, and were found to be cost-effective in reducing adverse pregnancy outcomes (22, 23). A recent study, showed that pre-pregnancy educational interventions in primary care over a period of four years, improved uptake of preconception folic acid and the achievement of an ideal glycaemic target for conception (24).

Our results expand the relevance of those findings, describing the patients who would benefit from these interventions with the breath of a 13-year observation of a nationally representative sample of patients (n = 6,657 in 2017) with diabetes attending primary care practices in England.

### Implication for Practice

In the general population, unplanned pregnancy is estimated to be around 40% (25). Preconception management of factors associated with negative pregnancy outcomes are likely to be beneficial for both mother and baby. National statistics demonstrated a decline of smokers in the last decades, as observed in this study (26), in women with diabetes. The increasing obesity rate remains to be effectively addressed also in women with a diagnosis of diabetes, especially in their reproductive age (27).

The adoption of 2015 NICE guidelines for diabetes and pregnancy increased the use of contraception in women with more than one unfavorable factors, especially in those with Type 1 diabetes (6). However more effective strategies for the improvement of pregnancy preparation in diabetes should implement active engagement about pregnancy planning and establish safe contraception where needed, as part of regular diabetes reviews. However, where women have no conception plans and/or are taking appropriate steps to avoid pregnancy, they should not be denied therapy that might improve the management of their diabetes and/or its complications. We considered all women not prescribed contraception at risk for unplanned pregnancy, similarly to the approach used in the London Measure of Unplanned Pregnancy (LMUP) (28) which includes contraception as one of six indicators for the definition of unintended pregnancy. There is no specific contraindication to contraception for women with diabetes and prescription guidance is the same as for healthy women, with the exception of women with advanced cardiovascular disease (29).

### Strengths and Limitations

The strength of our study lies in our real-world representative sample of women of reproductive age with diabetes in England. Data were collected from primary care centres and they reflect the reality of diabetes management, thus including also patients with milder forms of Type 2 diabetes who, nevertheless, do carry an increased risk for adverse pregnancy outcomes.

Limitations of the study are related to missing data for some variables of interest (e.g., BMI). Unfortunately, irregularities in the frequency of GP appointments and the limited time allocated to visit, may limit data collection and are beyond our control. However, the imperfect available data that we report conforms to what we would expect based on studies on similar patients in the UK (24).

Contraception may have been underestimated because the CMR system does not capture the use of contraceptives available without prescription.

### Future Research

Further research should focus on tailored educational interventions for both patients and healthcare professionals, as well as feedback aimed at improving the quality of care. Early data from MyPracticeDashboard, an innovative feedback platform, reports and compares recording rates of diagnoses and episodes for each practice of the RCGP RSC network (30). This type of interventions could be used to target women of childbearing age with diabetes from each practice, promoting continuous assessments of factors which would negatively influence pregnancy, not only glycaemic control, but also review of medications and lifestyles (31).

### Conclusions

From 2004 to 2017, several aspects of diabetes care improved for women with diabetes of childbearing age. However, most of these women have unfavorable factors for an unplanned pregnancy, especially women with Type 2 diabetes where the management involves the use of medications not recommended in the case of pregnancy. Pre-pregnancy care including more active promotion of family-planning, detailed medication review, and weight management should be enhanced in routine diabetes care to achieve safer pregnancy, wherever the care of these women takes place.

## Data Availability Statement

The datasets generated for this study can be found in the RCGP RSC repository. RCGP RSC data are available for the analysis following the application form that can be found online at www.rcgp.org.uk/rsc.

## Author Contributions

ND and SL conceived the idea for this study. MG contributed to the literature search, led the analysis, and wrote the manuscript. SL, ND, MF, MS, AC, and WH contributed to the literature search, the clinical discussion, and reviewed/edited the final manuscript. MG, MJ, and JVV contributed to the development of the statistical model and analysis. SL is the guarantor of this work and, as such, had full access to all the data in the study and takes responsibility for the integrity of the data and the accuracy of the data analysis. All authors contributed to the article and approved the submitted version.

## Funding

MG’s study at University of Surrey was supported by the European Union through the Erasmus exchange programme. This project had no external funding.

## Conflict of Interest

The authors declare that the research was conducted in the absence of any commercial or financial relationships that could be construed as a potential conflict of interest.

## References

[1] McGrogan A Snowball J de Vries CS. Pregnancy losses in women with Type 1 or Type 2 diabetes in the UK: an investigation using primary care records. Diabetes Med (2014) 31(3):357–65. 10.1111/dme.12332 24111989

[2] Wahabi HA Alzeidan RA Esmaeil SA. Pre-pregnancy care for women with pre-gestational diabetes mellitus: a systematic review and meta-analysis. BMC Public Health (2012) 12(1):792. 10.1186/1471-2458-12-792 22978747 PMC3575330

[3] England, Wales and the Isle of Man. 12th. 2017.

[4] Kulshrestha V Agarwal N. Maternal complications in pregnancy with diabetes. J Pak Med Assoc (2016) 66(9 Suppl 1):S74–7. 27582159

[5] Ludvigsson JF Neovius M Söderling J Gudbjörnsdottir S Svensson A-M Franzén S. Periconception glycaemic control in women with type 1 diabetes and risk of major birth defects: population based cohort study in Sweden. BMJ (2018) 362:k2638. 10.1136/bmj.k2638 29976596 PMC6031927

[6] National Insitute for Health and Care Excellence [NICE]. National Institute for Health and Care Excellence. Diabetes in pregnancy: management from preconception to the postnatal period (NG3). NICE Guideline [NG3] (2015) Vol. 63:42.

[7] Montouchet C Trussell J. Unintended pregnancies in England in 2010: costs to the National Health Service (NHS). Contraception (2013) 87(2):149–53. 10.1016/j.contraception.2012.06.008 PMC3496824 22878145

[8] American Diabetes Association AD. Management of Diabetes in Pregnancy: Standards of Medical Care in Diabetes-2018. Diabetes Care (2018) 41(Suppl 1):S137–43. 10.2337/dc18-S013 29222384

[9] de Lusignan S. Codes, classifications, terminologies and nomenclatures: Definition, development and application in practice. Inform Prim Care (2005). 10.14236/jhi.v13i1.580 15949178

[10] McGovern A Feher M Munro N de Lusignan S. Sodium-Glucose Co-transporter 2 (SGLT2) Inhibitor: Comparing Trial Data and Real-World Use. Diabetes Ther (2017) 8(2):365–76. 10.1007/s13300-017-0254-7 PMC5380507 28324484

[11] de Lusignan S Correa A Smith GE Yonova I Pebody R Ferreira F. RCGP Research and Surveillance Centre: 50 years’ surveillance of influenza, infections, and respiratory conditions. Br J Gen Pract (2017) 67(663):440–1. 10.3399/bjgp17X692645 PMC5604796 28963401

[12] Correa A Hinton W McGovern A van Vlymen J Yonova I Jones S. Royal College of General Practitioners Research and Surveillance Centre (RCGP RSC) sentinel network: a cohort profile. BMJ Open (2016) 6(4):e011092. 10.1136/bmjopen-2016-011092 PMC4838708 27098827

[13] Hinton W McGovern A Coyle R Han TS Sharma P Correa A. Incidence and prevalence of cardiovascular disease in English primary care: A cross-sectional and follow-up study of the Royal College of General Practitioners (RCGP) Research and Surveillance Centre (RSC). BMJ Open (2018). 10.1136/bmjopen-2017-020282 PMC6104756 30127048

[14] McGovern A Hinton W Correa A Munro N Whyte M de Lusignan S. Real-world evidence studies into treatment adherence, thresholds for intervention and disparities in treatment in people with type 2 diabetes in the UK. BMJ Open (2016) 6(11):e012801. 10.1136/bmjopen-2016-012801 PMC5168506 27884846

[15] Liyanage H Williams J Byford R Stergioulas L De Lusignan S. Ontologies in Big Health Data Analytics: Application to Routine Clinical Data. In: Studies in Health Technology and Informatics (2018). 30306908

[16] Liaw S-T Taggart J Yu H Lusignan S Kuziemsky C Hayen A. Integrating electronic health record information to support integrated care: Practical application of ontologies to improve the accuracy of diabetes disease registers. J BioMed Inform (2014) 52:364–72. 10.1016/j.jbi.2014.07.016 25089026

[17] Scavini M Rossi MC Scardapane M Nicolucci A Manicardi V Russo G. Portrait of women with type 1 or type 2 diabetes of childbearing age attending diabetes clinics in Italy: the AMD-Annals initiative. Acta Diabetol (2018) 55(2):193–9. 10.1007/s00592-017-1076-9 29209815

[18] Health Research Authority. Is my study research? Available ar: http://www.hra-decisiontools.org.uk/research/.

[19] Murphy HR Bell R Cartwright C Curnow P Maresh M Morgan M. Improved pregnancy outcomes in women with type 1 and type 2 diabetes but substantial clinic-to-clinic variations: a prospective nationwide study. Diabetologia (2017) 60(9):1668–77. 10.1007/s00125-017-4314-3 PMC5552835 28597075

[20] Casson IF. Pregnancy in women with diabetes-after the CEMACH report, what now? Diabetes Med (2006) 23(5):481–4. 10.1111/j.1464-5491.2006.01896.x 16681556

[21] Makda SI Davies MJ Wilmot E Bankart J Yates T Varghese EM. Prescribing in pregnancy for women with diabetes: use of potential teratogenic drugs and contraception. Diabetes Med (2013) 30(4):457–63. 10.1111/dme.12051 23110381

[22] Murphy HR Roland JM Skinner TC Simmons D Gurnell E Morrish NJ. Effectiveness of a Regional Prepregnancy Care Program in Women With Type 1 and Type 2 Diabetes: Benefits beyond glycemic control. Diabetes Care (2010) 33(12):2514–20. 10.2337/dc10-1113 PMC2992180 21115765

[23] Egan AM Danyliv A Carmody L Kirwan B Dunne FP. A Prepregnancy Care Program for Women With Diabetes: Effective and Cost Saving. J Clin Endocrinol Metab (2016) 101(4):1807–15. 10.1210/jc.2015-4046 26918293

[24] Yamamoto JM Hughes DJF Evans ML Karunakaran V Clark JDA Morrish NJ. Community-based pre-pregnancy care programme improves pregnancy preparation in women with pregestational diabetes. Diabetologia (2018) 61(7):1528–37. 10.1007/s00125-018-4613-3 PMC6445478 29744539

[25] Singh S Sedgh G Hussain R. Unintended pregnancy: worldwide levels, trends, and outcomes. Stud Fam Plann (2010) 41(4):241–50. 10.1111/j.1728-4465.2010.00250.x 21465725

[26] Adult smoking habits in England - Office for National Statistics. . https://www.ons.gov.uk/peoplepopulationandcommunity/healthandsocialcare/healthandlifeexpectancies/bulletins/adultsmokinghabitsingreatbritain/2019#:~:text=The.

[27] Stephenson J Heslehurst N Hall J Schoenaker DAJM Hutchinson J Cade JE. Before the beginning: nutrition and lifestyle in the preconception period and its importance for future health. Lancet (2018) 391(10132):1830–41. 10.1016/S0140-6736(18)30311-8 PMC6075697 29673873

[28] Hall JA Barrett G Copas A Stephenson J. London Measure of Unplanned Pregnancy: guidance for its use as an outcome measure. Patient Relat Outc Meas (2017) 8:43–56. 10.2147/PROM.S122420 PMC5388237 28435343

[29] Barrett G Smith SC Wellings K. Conceptualisation, development, and evaluation of a measure of unplanned pregnancy. J Epidemiol Community Heal (2004) 58(5):426–33. 10.1136/jech.2003.014787 PMC1732751 15082745

[30] Pathirannehelage S Kumarapeli P Byford R Yonova I Ferreira F de Lusignan S. Uptake of a Dashboard Designed to Give Realtime Feedback to a Sentinel Network About Key Data Required for Influenza Vaccine Effectiveness Studies. Stud Health Technol Inform (2018) 247:161–5. 29677943

[31] Nwolise CH Carey N Shawe J. Exploring the acceptability and feasibility of a preconception and diabetes information app for women with pregestational diabetes: A mixed-methods study protocol. Digit Heal (2017) 3:205520761772641. 10.1177/2055207617726418 PMC6001229 29942610

