# Supplementary material for: Trends in Factors Affecting Pregnancy Outcomes Among Women With Type 1 or Type 2 Diabetes of Childbearing Age (2004–2017)
Source: Front Endocrinol (Lausanne). 2021 Feb 22;11:596633. doi: 10.3389/fendo.2020.596633 (PMC7937966; doi:10.3389/fendo.2020.596633)
Supplement: Supplementary file 1 [file Table_1.docx]

**Supplementary Table**

**Supplementary Table 1:** Description of RCGP RSC cohort over the period 2004 to 2017 with prevalence of women of childbearing age with diabetes.

| **Year** | **Total patient in RCGP RSC** | **Total women of RCGP RSC**  **n(%)** | **Women of childbearing age**  **n(%)** | **Women of childbearing age with diabetes**  **n (%)** | **Women of childbearing age with type 2 diabetes**  **n (%)** |
| --- | --- | --- | --- | --- | --- |
| **2004** | 1489929 | 760145  (51.0) | 316461  (41.6) | 3218  (1.0) | 1978  (61.5) |
| **2005** | 1541630 | 783657  (50.8) | 325961  (41.6) | 3457  (1.1) | 2149  (62.2) |
| **2006** | 1601940 | 813632  (50.8) | 337712  (41.5) | 3747  (1.1) | 2364  (63.1) |
| **2007** | 1654056 | 839529  (50.8) | 348531  (41.5) | 3997  (1.1) | 2542  (63.6) |
| **2008** | 1692880 | 858998  (50.7) | 355892  (41.4) | 4271  (1.1) | 2747  (64.3) |
| **2009** | 1741569 | 882396  (50.7) | 364473  (41.3) | 4479  (1.2) | 2899  (64.7) |
| **2010** | 1799643 | 911241  (50.6) | 375600  (41.2) | 4752  (1.2) | 3082  (64.9) |
| **2011** | 1856211 | 939662  (50.6) | 387099  (41.2) | 5001  (1.3) | 3261  (65.2) |
| **2012** | 1916444 | 970551  (50.6) | 400526  (41.3) | 5322  (1.3) | 3495  (65.7) |
| **2013** | 1997193 | 1012811  (50.7) | 421774  (41.6) | 5725  (1.4) | 3742  (65.4) |
| **2014** | 2070953 | 1052097  (50.8) | 442416  (42.1) | 6023  (1.4) | 3916  (65.0) |
| **2015** | 2118851 | 1075375  (50.8) | 453088  (42.1) | 6297  (1.4) | 4086  (64.9) |
| **2016** | 2154817 | 1094003  (50.8) | 460991  (42.1) | 6509  (1.4) | 4226  (64.9) |
| **2017** | 2186003 | 1109602  (50.8) | 465898  (42.0) | 6657  (1.4) | 4325  (65.0) |
